# The differential effects of a complex protein drink versus isocaloric carbohydrate drink on performance indices following high-intensity resistance training: a two arm crossover design

**DOI:** 10.1186/1550-2783-10-31

**Published:** 2013-06-12

**Authors:** Shannan Lynch

**Affiliations:** 1 Trident University, 5757 Plaza Drive #100, Cypress, CA, USA

**Keywords:** Isocaloric carbohydrate, Protein drink/beverage, Performance, Repeated-bout, Nutrient timing

## Abstract

**Background:**

Post-workout nutrient timing and macronutrient selection are essential for recovery, glycogen replenishment and muscle protein synthesis (MPS). Performance repeatability, particularly after strenuous activity, can be influenced by substrate availability, recovery markers and perceived rate of exertion. This study compared the differential effects of a complex protein ready-to-drink beverage (VPX) and isocaloric carbohydrate beverage (iCHO) on performance—agility T-test, push-up test, 40-yard sprint, and rate of perceived exertion (RPE), following high-intensity resistance training (HIRT).

**Methods:**

In a randomized, double blind two-arm crossover controlled trial, 15 subjects performed a 15–18 minute (2:1 work to rest) HIRT and then immediately drank one of the two treatments. After a 2-hour fast, subjects returned to execute the field tests and report RPE. The protocol was repeated one week later with the other treatment.

**Results:**

There were no significant main effect differences in the agility T-test (p = 0.83), push-up (p = 0.21) sprint (p = 0.12), average agility RPE (p = 0.83), average push-up RPE (p = 0.81) or average sprint RPE (p = 0.66) between the two trials and the two treatments. The multivariate analysis yielded a cumulative significant interaction effect amongst the three performance variables after consuming VPX (p < 0.01). These results suggest a complex protein beverage is a better post-workout choice compared to an isocaloric carbohydrate beverage for repeated performance for activities that require multiple energy demands and athletic skills; however, this outcome was not observed for each single performance event or RPE.

**Conclusion:**

When considering the collective physical effects of the agility T-test, push-up and sprint tests, a complex protein beverage may provide a recovery advantage as it relates to repeated-bout performance compared to an iCHO-only beverage. Additional research examining the chronic effects of post-exercise protein versus iCHO beverages on performance repeatability, particularly in special populations (e.g. tactical and elite athletes), is warranted to further develop these findings.

## Background

Applying the science of nutrient timing, this study examined the differential effects of two beverages—a ready-to-drink 1:4 carbohydrate to protein beverage (VPX) and an isocaloric carbohydrate powdered beverage (iCHO)—on exercise performance indices and rate of perceived exertion (RPE) following high-intensity resistance training (HIRT). Post-exercise, it appears there is a plastic window of opportunity to efficiently replenish glycogen and support the processes of repair and stimulate muscle protein synthesis (MPS). Refueling after exercise, ideally within 30 minutes and no more than two hours, has been shown to positively influence the repletion of glycogen stores and augment protein synthesis [1]. Although the nutrient timing theory has been challenged and recent evidence argues that multiple factors can influence the rationale of the “window of opportunity” [2], the strategy for immediate post-exercise re-feeding is applicable to activities that require multiple bouts and/or glycogen-depleting endurance events [3]. Carbohydrate and protein drinks are leading sources for post-exercise refueling due to their absorptive properties, but there is disagreement as to which of the two macronutrients are most effective post-workout, specifically as it relates to nutrient timing and supporting recovery.

The study product contains an approximate 1:4 ratio of carbohydrate (CHO) to protein (PRO), and claims to provide a fast system for replenishment to promote protein synthesis and recovery via a low sugar, high-protein complex and vitamin-fortified blend. High-intensity resistance training involves eccentric exercises that may elevate inflammatory markers, instigate damaging morphological changes, decrease subsequent performance, deplete muscle glycogen, increase indicators of muscle damage (e.g., elevated creatine kinase and myoglobin) and inflammatory constituents (e.g., high-sensitivity C-reactive protein) [4-9]. In addition to physiological alterations, exhaustive exercise (such as HIRT) can disturb successive fitness/ athletic performance [10,11]. Most sports and physically taxing situations, such as tactical operations (i.e., police, fire or military), require the individual to repeat performance efforts such as speed, agility and muscular endurance. Sports and tactical specific conditioning can groove the neuromuscular and physical demands, but post-workout nutrition is imperative to support metabolic repair and nutrient requirements, especially for activities that require multiple daily workouts (“two-a-days”) or repeated bouts of exertion.

Muscle recovery and glycogen replenishment are two chief concerns related to post-exercise nutrition needs, especially after high-intensity exercise such as resistance training and interval-based activities. The damaging effects of exercise create a need for effective post-workout nutrition to replenish glycogen and boost protein synthesis [1,12]. Fitness and sports settings are notable areas of research on this topic, but muscle recovery and re-synthesis are as equally important to other fields that require physically stressful conditions. The effects of high-intensity, glycogen-depleting exercise on subsequent activity—especially in athletic and tactical environments—pose a potential concern for recovery and performance ability. The previous effects of dietary interventions and nutrient timing, such as amino acid [2,13], carbohydrate, and protein consumption [3,14,15] on exercise recovery validates the importance of post-exercise feeding.

The goal of this study was to compare two supplement beverage products and determine their relative effects on fitness performance indices (agility T-test, push-up test, and 40-yard sprint) following exhaustive exercise. In addition, the design incorporated a scaled component to measure the rate of perceived exertion (RPE) between the two interventions [16,17]. Although comparing two products is not a novel concept to date, no one has tested a ready-to-drink commercially manufactured complex protein drink with an isocaloric CHO drink against this methodology, and the exercise portion is unique because the workout requires subjects to complete a total body HIRT workout prior to executing the outcome measures; opposed to executing single joint, isolated exercises in a laboratory setting. The workout actually mimicked the fatigue experienced in a total body resistance training session or exhaustive physical bout. Finally, the existing literature reports inconclusive outcomes in regards to the efficacy of PRO-CHO, PRO-only, and CHO-only supplements on post-exercise performance and recovery [1,13,18-20]. Considering these inconsistencies, expanded research to understand these discrepancies is needed. Although many sources agree that immediate or within 30 minutes post-exercise re-feeding is a plastic time frame for glycogen-depleting and/or multiple bout events [1,3,21], complex PRO versus an iCHO supplementation is not consistently understood in regards to its role on recovery and subsequent activity. Accordingly, more information is needed to expand upon this area of sports nutrition and clarify which substrates are most effective in the post-exercise state for repeat performance.

## Methods

### Subjects and screening

Fifteen male subjects (31.7 ± 6.2 yrs old) were randomly recruited from a fitness center in Burbank, CA (at the time the facility had approximately 700 members). To recruit, an email flyer was sent to all male members who fell between the ages of 21 and 44 years of age. In addition, the flyer was posted in the facility two weeks prior to the start of the study to generate a list of interested volunteers. Men who responded to the advertisements were emailed a screening form. All subjects had to be categorized as “low risk” according to the American College of Sports Medicine [22] and have been exercising at least five times per week for at least an hour for a year or more, and have at least one year of strength training experience. Subjects had a combination of exercise history; all subjects participated in a variety of cardiovascular (e.g. jogging and/or indoor cycling classes), interval training (group fitness classes) and resistance training (weight room training). Subjects were excluded if they had any musculoskeletal conditions that limited their ability to complete the physical requirements and/or had any dietary limitations that affected their ability to participate.

The Trident University Institutional Review Board approved this study to be in ethical compliance for human trials and identified the level of review as “minimal risk” based on the evaluation that the conditions do not exceed the subjects’ daily ordinary risks and that the interests of the subjects are protected. The VPX Protein Rush™ Chocolate Dream product was donated by the manufacturer. The researcher has no conflicting relationships with manufacturer, and no further benefits have been provided as a result of the manufacturer’s product donation. This study was conducted with no commercial bias or benefits to the investigator throughout the duration of the investigation.

### Design

**Figure 1:**
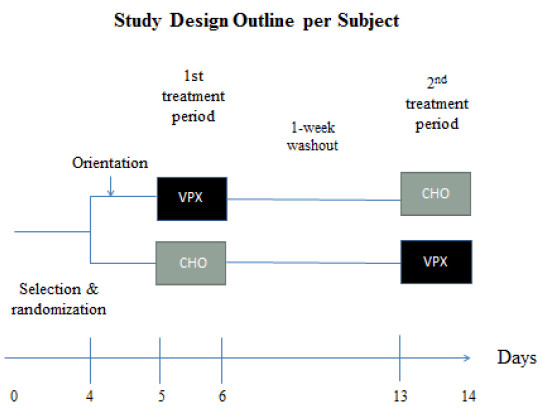
**Study design outline per subject.** The research design outline provides a timeline depicting the commitment duration per subject. Overall, the total duration of the study lasted 14 days for each subject. Both treatment arms took place on a single day with a 1-week washout in between.

A randomized, two-arm crossover trial with a 1-week wash-out period was employed. Each arm lasted one day per subject, and subjects were tested on the same day of the week and time of day for each arm. Each subject was asked to attend a familiarization and 10RM determination session no more than a week prior to testing. A 24-hour dietary and activity recall was collected the day of each trial to monitor subjects’ activity and dietary consumption trends prior to each arm. Each subject began the trial with a 10 min standardized, dynamic warm-up; thereafter subjects executed the following high-intensity resistance training workout for 2 min, for as many rounds as possible, followed by 1 min of rest for 5–6 sets: (with a 25% overhead push-press 1-repetition maximum (RM) 8 - dumbbell push-press → 8 - squats (dumbbells at sides) → 8 - dumbbell push-ups → repeat until rest period. The average number of rounds (and consequently repetitions) per set were counted to evaluate volume consistency. Within 5 minutes of completing the workout, subjects were randomly assigned to ingest one of the two beverage interventions—VPX Protein Rush™ Chocolate Dream or concentrated isocaloric Gatorade® orange flavor (see Table 1 for beverage nutrient composition)—and then the subjects returned two hours later to the testing location to execute the performances tests and report RPE. Subjects did not consume anything except water between the HIRT workout and the performance tests (2-hour fast). The second arm was repeated after a 1-week wash-out with the other intervention. Overall, the entire trial lasted 14 days. See Figure 1 for the schematic.

**Table 1:** Beverage composition

**Nutrient breakdown**	**VPX (17 fl. oz)**	**iCHO (20 fl. oz)**
Total calories	260	260
Calories from Fat	55	0
Carbohydrate (g)	11	68^a^
Sugars (g)	6	68
Cholesterol (mg)	25	0
Total fat (g)	6	0
Saturated fat (g)	1.5	0
Protein (g)	40	0
Sodium (mg)	380	540
Potassium (mg)	-	150

^a^iCHO manufacturer lists their product as 68 g of CHO and 260 calories; however 68 g of CHO equals 272 calories according to the assumption that CHO contains four calories per gram.

### Data collection

**Figure 2:**
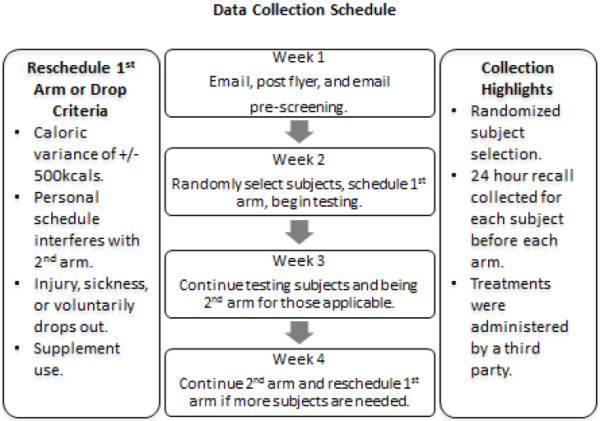
**Data collection schedule.** The above figure depicts the data collection timeline and collection details. Subjects committed for a period of eight consecutive days for data collection and provided a 24 hour diet and exercise recall.

Subjects’ anthropometric data (weight and height) was collected and recorded by the principal investigator using a calibrated Omron HBF-400 body weight scale (Omron, Bannockburn, IL) and a wall-mounted Seca 206 stadiometer (KWS Medical, North Bend, WA). The 1RM push-press load was estimated by conducting the 10RM estimation protocol [23] to calculate the 25% 1RM. The 40-yard sprint and agility T-test distances were measured using a measurement wheel (Keson, Aurora, IL) and timed using an Accusplit S3MAGXLBK stopwatch (Accusplit, Livermore, CA) and basic athletic cones. The push-up test was measured based on the subjects’ to-fatigue maximum repetition. The RPE scale was measured using a previously validated tool—the 15-point Borg scale [17]. The 24-hour diet and activity recalls were collected to determine typical dietary intakes and activity trends using Fitday.com® (Internet Brands®, El Segundo, CA) [24]. A nutritionist analyzed their variances. Subjects were asked not to change their typical dietary or activity habits during the trial period, and to mimic their diet and activity habits prior to each trial. Refer to Figure 2 for schedule details.

### Statistical analysis

Statistical analysis was performed using SPSS 18 (IBM, Armonk, NY). Data were analyzed by a repeated-measures analysis of variance (RM-ANOVA) to detect any significant effects for product, trial, and product*trial effects between the beverages and the performance tests and RPE. Covariates (HIRT repetitions and 24-hour caloric intake and energy expenditure) were considered; however, since the HIRT variance was zero and the caloric variance did not exceed ±500 calories, they were excluded from the statistical analysis. In addition, a repeated-measures multivariate analysis of variance (RM-MANOVA) was analyzed to detect any significant interaction effects between product*trial*tests (agility*push-up*sprint). A paired t-test (two levels) was used to determine significant differences between within-subject performance tests and RPE [25]. A full descriptive analysis was generated. A p-value of < 0.05 was considered significant.

## Results

### Subject descriptives

Subjects were similar in age (31.73 ± 6.24 years) and height (1.76 ± 0.073 m). Weight and BMI reported more variability amongst the measures of central tendency. Despite this wide variance, all subjects met the inclusion criteria for the study. See Table 2 for subject descriptive characteristics.

**Table 2:** Subject descriptive statistics

**Demographics**	**Mean**	**SD**
Age - years	31.73	6.24
Height - m	1.76	0.073
Weight - kg	80.50	16.45
BMI- kg/m^2^	26.22	5.96

### HIRT and caloric intake variance

Table 3 presents two of the controlled factors—HIRT repetitions and calorie consumption between the two arms. As a control, subjects were required to stay within 10% of the repetitions completed in trial 1. There were no variances in HIRT repetitions between the two trials because the study team kept the subjects on tempo to achieve the same number of repetitions as they did the previous week. A paired t-test was used to determine the pooled difference of caloric means between trial 1 and trial 2. Subjects’ 24-hour caloric consumption prior to trial 1 (2,346.9 ± 114.0 kcals) was not significantly different compared to their 24-hour caloric consumption prior to trial 2 (2,302.9 ± 134.6 kcals, p = 0.58). Therefore, the HIRT and 24-hour caloric consumption were not threats to validity based on this investigation’s parameters.

**Table 3:** High-intensity resistance training (HIRT) and calorie variances for trials 1and 2

**HIRT-total repetitions/ subject**			**24-hour calorie consumption recall-Kcals/subject**		
**Trial 1**	**Trial 2**	**Variance**	**Trial 1**	**Trial 2**	**Variance**
384	384	0	2,718	2,457	261
488	488	0	1,737	1,314	423
463	463	0	2,462	2,289	173
372	372	0	2,960	3147	−187
370	370	0	2,422	2035	387
350	350	0	1,785	2148	−363
401	401	0	2,450	2865	−415
469	469	0	2,577	2765	−188
321	321	0	1,968	2204	−236
305	305	0	2,805	2660	145
374	374	0	1,545	1209	336
442	442	0	2,187	2340	−153
346	346	0	2,659	2226	433
347	347	0	2,078	2234	−156
339	339	0	2,851	2651	200

### Performance and RPE

Table 4 presents the paired sample statistics for performance and RPE. The largest variances were seen in the push-up performance test and push-up RPE. However, according to the paired sample t-tests (Table 5) the results indicate no significant mean differences between VPX and iCHO. The variable closest to reporting a significant finding was the mean difference between sprint time (VPX = 5.91 ± 0.57 seconds; iCHO = 5.77 ± 0.53 seconds [p = 0.12]).

**Table 4:** Paired samples statistics for the performance tests and rate of perceived exertion

**Variables**		***M***	***N***
^**a**^Pair 1	VPX Agility	12.9	15
	iCHO Agility	12.8	15
^**b**^Pair 2	VPX Push-up	49.40	15
	iCHO Push-up	51.93	15
^**a**^Pair 3	VPX Sprint	5.91	15
	iCHO Sprint	5.77	15
^**c**^Pair 4	VPX Agility RPE	13.90	15
	iCHO Agility RPE	14.02	15
^**c**^Pair 5	VPX Push-up RPE	15.33	15
	iCHO Push-up RPE	15.20	15
^**c**^Pair 6	VPX Sprint RPE	15.73	15
	iCHO Sprint RPE	15.53	15
^**c**^Pair 7	Average RPE VPX	15.28	15
	Average RPE iCHO	14.81	15

^a^Measured in secconds. ^b^Measured in repetitions. ^c^Scale of 6–20.

**Table 5:** Paired samples t-test for the performance tests and rate of perceived exertion

**Paired differences**						
			**95% CI of the difference**			
**Variables**	***M***	***SD***	**Lower**	**Upper**	***t*(14)**	**p-value (2-tailed)**
^**a**^Agility VPX-iCHO	0.04	0.76	−0.38	0.46	0.22	0.83
^**b**^Push-up VPX-iCHO	−2.53	7.50	−6.69	1.62	−1.31	0.21
^**a**^Sprint VPX-iCHO	0.14	0.32	−0.04	0.31	1.66	0.12
^**c**^RPE Agility VPX-iCHO	−0.12	2.00	−1.23	0.99	−0.23	0.83
^**c**^RPE Push-up VPX-iCHO	0.13	2.13	−1.05	1.31	0.24	0.81
^**c**^RPE Sprint VPX-iCHO	0.20	1.73	−0.76	1.16	0.45	0.66
^**c**^RPE Average VPX-iCHO	0.47	1.33	−0.27	1.20	1.36	0.19

CI = confidence interval. ^a^Measured in secconds. ^b^Measured in repetitions. ^c^Scale of 6–20.

The RM-ANOVA determined the separate univariate effects. The RM-ANOVA assessed if there were any significant effects in the dependent variables between the two trials (time) and if there was a significant interaction between the time and treatment. None of the RM-ANOVA yielded singular, main effects for any of the performance or RPE tests such that the mean measurement was not significantly different for VPX than for iCHO (Tables 6 and 7).

**Table 6:** RM-ANOVA of within-subjects contrasts for performance tests

**Source**	**Measure**	**Time**	**df**	***F***^**a**^	**p-value**	**Observed power**^**b**^
**Time**	**Agility**	**Linear**	**1**	**0.049**	**0.83**	**0.06**
	Pushup	Linear	1	1.71	0.21	0.23
	Sprint	Linear	1	2.77	0.12	0.34
Error (Time)	Agility	Linear	14			
	Pushup	Linear	14			
	Sprint	Linear	14			

^a^Geisser/Greenhouse correction. ^b^Computed using alpha = 0.05.

**Table 7:** ANOVA of within-subjects contrasts for rate of perceived exertion

**Source**	**Measure**^**c**^	**Time**	**df**	**Mean square**	***F***^**b**^	**p-value**	**Observed power**^**a**^
Time	Agility RPE	Linear	1	0.10	0.05	0.83	0.06
	Push-up RPE	Linear	1	0.13	0.06	0.81	0.06
	Sprint RPE	Linear	1	0.30	0.20	0.66	0.07
Error (Time)	Avg RPE	Linear	1	1.63	1.86	0.19	0.25
Error (Time)	Agility RPE	Linear	14	2.00			
	Push-up RPE	Linear	14	2.23			
	Sprint RPE	Linear	14	1.50			
	Average RPE	Linear	14	0.88			

^a^Computed using alpha = 0.05. ^b^Geisser/Greenhouse correction. ^c^Scale of 6–20.

Lastly, a repeated-measures multivariate analysis (RM-MANOVA) was used to simultaneously test each treatment’s interaction effect on the performance tests. The RM-MANOVA yielded a significant interaction effect for the three performance variables (p < 0.01). Therefore, the null hypothesis that there is no significant difference on performance when comparing the effects of VPX versus iCHO on performance following HIRT can be rejected. There was a significant interaction effect between the agility T-test, push-up, and sprint tests indicating the performance effect of VPX on the three performance tests—when considered collectively—was greater than iCHO. Table 8 reports the RM-MANOVA results. A RM-MANOVA for RPE was not analyzed because the interaction effect for the average RPE for each treatment was sufficiently assessed in the univariate analysis.

**Table 8:** Results of the RM-MANOVA of within-subjects contrasts for performance tests

**Effect**			**Value**	***F***^**a**^	**p-value**	**Observed power**^**b**^
Within subjects	Time	Wilks’ Lambda	0.30	9.17	0.002	0.97

## Discussion

The purpose of this study was to examine the differential effects of a complex protein beverage and an isocaloric CHO beverage on performance measures and RPE following high-intensity resistance training.

High-intensity exercise—especially high-intensity resistance training—can significantly deplete muscle glycogen. Towards the end of the 15–18 minute 2:1 work to rest HIRT workout all subjects were experiencing cardiovascular and muscular fatigue. This HIRT workout was an original protocol developed by the primary researcher. However, it was inspired by previous studies that measured performance and/ or recovery following ingestion or supplementation of treatments such as Smith et al. [26] who utilized a 15–18 minute high-intensity cycling protocol to glycogen dilute the legs. The current design required subjects to whole-body glycogen dilute by executing compound, total body resistance and body weight exercises in a continuous, explosive pattern for two minutes. Most subjects could not reach 18 minutes (most stopped at 15 minutes) due to exhaustion; thus, implying the protocol was physically taxing and adequate to glycogen-deplete the muscles and instigate catabolic processes.

In addition, the mechanical stress associated with resistance training places eccentric loading forces on the muscle fibers during muscle contraction, which micro-tears the muscle, and this catabolic environment hosts the mechanisms that affect MPS [12,27]. Theoretically, the consumption of macronutrients and the timing of such could affect the neuromuscular response to exercise by counteracting the negative physiological state that follows. The present investigation demonstrated that a beverage, primarily comprised of protein (approximately a 1:4 CHO to PRO ratio), provides better post-exercise replenishment for subsequent agility T-test, push-up, and sprints tests compared to an iCHO-only drink. These practical field tests were used to assess physical ability, not clinical presentations. However, the outcomes of this study can be explained by mechanisms supported in other research that utilized more invasive protocols and designs. For example, nuclear magnetic resonance spectroscopy (nMRS) is a widely used clinical tool for the observation of high-energy phosphates, such as glycogen. The technique is a minimally invasive procedure that permits in-vivo, time-dependent information to be evaluated [28]. Ivy et al. [29] utilized nMRS as a method to evaluate glycogen content within the vastus lateralis pre-exercise and four hours post-exercise. These findings suggested that consuming a CHO-PRO supplement compared to a CHO-only supplement may replenish muscle glycogen more effectively post-exercise. This information is transferable to the current study because carbohydrate availability and MPS are important for post-exercise recovery and subsequent performance. Replenishing muscle glycogen content after exercise is crucial to mitigate tissue damage, inflammatory markers, and upregulate the Akt/PKB pathway for MPS. The focus of the current study was to evaluate the performance and RPE differences between two products by conducting physical tests and reporting exertion. In other words, regardless of muscle glycogen content, the interest lied within the subjects’ ability to perform and which treatment provided the substrates to do so. Since glucose availability is necessary for glycogen synthesis, the objective was to indirectly determine which treatment (VPX or iCHO) provided the best substrate for glycogen synthesis, (and by conjunction recovery and repeated performance), whether it be through glucose-mediated glycogenesis or gluconeogenesis.

Macronutrient selection and recovery are indecisive topics within the sports nutrition field. Some experts back the CHO-only recovery supplement, while others stand by the 4:1 ratio of CHO to PRO, and then some advocate PRO-only. VPX Protein Rush™ falls somewhere in the middle with its proprietary mix of: calcium caseinate, milk protein isolate, whey protein concentrate, micellar casein, whey protein isolate, casein hydrolysate di- and tri-peptides, and whey protein hydrolysate di- and tri-peptides. It contains 11 g of CHO, with 6 g attributing to dietary fiber, which is a considered “non-impact” CHO because fiber does not contribute to caloric content or affect blood glucose levels and insulin response. Net protein balance and muscle accretion are essential to performance, and following physical activity, negative protein balance supersedes protein synthesis until consumption of amino acids occurs [30]. Bovine milk protein contains approximately 80% casein and 20% whey [31,32]. Known as the “slow-releasing” protein, casein acts as an inhibitor to whole body protein breakdown, by means of sustaining whole body leucine balance, which is the critical amino acid for MPS [33]. However, casein is not a major contributor to new muscle accretion; rather it digests slowly to prevent the breakdown of existing muscle and preserves leucine balance. VPX also contains whey protein isolate, which is higher in quality compared to whey protein concentrate. When combined with resistance training, whey protein isolate has been shown to result in significantly greater gains in lean mass and strength compared to casein [34].

In regards to recovery for subsequent performance, the aim is to stunt muscle glycogen loss and catabolism while augmenting glycogen repletion and MPS, which entails replenishing lost muscle glycogen stores (which was discussed earlier), stimulating muscle recovery pathways, and reducing inflammatory and catabolic constituents. VPX possesses both glycogenic and anabolic characteristics to support the goals of recovery. Despite the small amount of CHO, the drink composition offers the qualities of fast-acting and slow-releasing proteins. Dietary protein is necessary to activate the MPS pathway, specifically mammalian target of rapamycin that signals initiation factors (p70S6K and 4EBP) responsible for activating messenger RNA translation initiation and ribosomal activity, which are rate-limiting steps for controlling protein synthesis. Catabolic factors, such as cortisol, creatine kinase, and lactate dehydrogenase, are detrimental to positive net protein balance. Neither hormone or enzyme profiles were assayed for this dissertation, but preceding investigations [13,35] measured hormonal profiles and catabolic markers, including testosterone, cortisol, creatine kinase, and lactate dehydrogenase. The current study connects to these outcome measures because adequate and timely post-exercise replenishment is intended to reduce catabolic and inflammatory markers and improve repeated performance; thus the performance tests in this study were practical extensions of the aforementioned clinical tests.

Although the present investigation measured short-term performance effects of the beverages, the blend of proteins in VPX contains the amino acids that potentially support muscle protein synthesis, recovery, and performance compared to the iCHO. Additionally, the smaller whey hydrolysate di- and tri-peptides—which are quickly digested—have the potential to be used as gluconeogenic substrates to replenish glycogen. Especially in a depleted state, some amino acids (i.e., alanine) can be used as a substrate to manufacture glucose.

Finally, as it pertains to the primary research hypothesis—following a high-intensity resistance workout, there is a differential effect on subsequent agility T-test, push-up test, and 40-yard sprint when supplementing post-workout with VPX versus an iCHO drink. Particularly, VPX yielded a significantly larger interaction effect between the performance tests following HIRT compared to iCHO. Repeated performance is a combined series of effort (often entailing more than one exercise modality and/or skill); hence, it is important a product has collective benefits rather than just improving one measure.

### Macronutrient and rate of perceived exertion

Exertion levels, or even “perceived” exertion levels, during exercise may affect performance. Very few studies have investigated the effects of PRO alone on RPE. The investigations by Backhouse et al. [36,37] supported the supplementation of CHO to lower RPE during exercise. Kalman [38] compared the effects of CHO-only, PRO-CHO, and PRO-only on various performance measures (i.e. resistance training), including RPE. The results did not report a significant difference in RPE between groups over time. This study reported similar findings with respect to differences between means and hypothesis testing via ANOVA—neither treatment was statistically significant towards reducing agility T-test, to-fatigue push-up, or 40-yard sprint RPE following HIRT.

Rate of perceived exertion is a subjective measurement, and studies by Utter et al. [39-42] that examined the effects of CHO on RPE observed that RPE does not correlate with the amount of total work actually performed. Subjects may have “felt” more fatigued after consuming a placebo compared to CHO, but there were no mean differences in performance between groups. Similarly, the current investigation found VPX and iCHO to be equivocal in terms of the subjects’ reported RPE; in other words, this is the first study to find that VPX provides similar exertion responses to an iCHO drink.

### Limitations

The ANOVA and t-test statistical results were not significant for any individual dependent variables. This could have been attributed to sample size and power (80%). The RM-MANOVA was not affected by the sample size and resulted in a meaningful and significant difference; this model reported a significant cumulative effect between the three performance tests. This outcome is likely attributed to the similarities between the tests (i.e., exercise performance variables) and their collective impact; as the variables were added into the model their compounded effects on each other became statistically apparent. Physical activity is a cumulative action often involving a combination of endurance, speed, agility, power and balance to name a few. It may be valuable to see cumulative effects than singular effects in exercise performance for athletes and exercisers who rely on more than one energy system and skill to complete a task or activity. Beyond the statistical limitations, state anxiety appeared to be a limitation for all subjects.

It is possible the subjects had apprehension leading into the second workout test. As a result, to preserve energy almost all subjects may have started slower during the first set of the second arm; therefore, subjects required verbal coaching to stay on task. Measuring pre- and post-glycogen status was not feasible for this design; however, subjects were asked to eat similar food composition before each arm. Lastly, despite each subject acting as their own control, the inclusion of an isolated control group (no treatment) would have provided an additional comparison to evaluate the effects of re-feeding versus no re-feeding. Particularly for the RPE hypothesis, which resulted in no differences between means, including an isolated control group could have provided data to support the importance of re-feeding to reduce RPE. For this particular study design, including a control group could have been unethical considering the setting and absence of medical personnel.

## Conclusions

The findings of this study are important to the sports and exercise performance industries because there is a need for novel research on specific macronutrient products. The outcomes exploit the benefits of consuming a complex protein drink versus carbohydrate-only beverage following glycogen depleting exercise. In addition, the 2:1 HIRT protocol (which is was an original design created by the primary researcher) could be used in other nutrient timing or performance studies as a tool to measure performance or implemented in a similar capacity, i.e. glycogen depleting exercise.

Nutrition experts are frequently asked to recommend specific products for supplementation, and this design used VPX Protein Rush™ and concentrated Gatorade®, two products that are accessible to the public. This study elucidated the differential effects of a ready-to-drink, complex protein beverage and an iCHO-only beverage on common performance measures, and offers practical information on nutritional post-workout strategies to prepare for repeated performance.

Controlled studies within the sports medicine and exercise performance fields provide valuable insight into how the human body reacts to and recovers from high intensity physical exercise. Results from this, and other similar studies can be beneficial when applied to high stress, intense performance professions, such as firefighters, disaster relief workers and the military. The use of protein supplements during prolonged physical effort can be an invaluable source of energy when endurance is critical. This study strongly indicates that after intense activity, consumption of a complex protein beverage may favorably influence subsequent physical performance better than an isocaloric carbohydrate drink. Based on this information, complex protein beverages may provide advantages to individuals with acute physical stressors as well as tactical operators and high performance athletes. Additional research is warranted.

## Abbreviations

1RM: Maximum weight one can lift safely one time; 25% 1RM: 25% of one’s 1 repetition maximum; CHO: Carbohydrate; Exhaustive exercise: Type of exercise intended to push individual beyond aerobic and lactate thresholds with the purpose of depleting glycogen and bringing them to a point of volitional fatigue; High-intensity resistance training (HIRT): High-intensity resistance training is a form of exhaustive exercise that has been shown to deplete muscle glycogen and increase indicators of muscle damage. Modality uses characteristics of HIIT, but with external resistance as a means to load muscle and induce muscle protein synthesis while improving strength and power; iCHO: Isocaloric carbohydrate; MPS: Muscle protein synthesis; Performance: Physical achievement; PRO: Protein; Rep: Repetition in regards to weight lifting; Rounds: Groups of sets; RPE: Rate of perceived exertion; Set: Group of repetitions; Volitional fatigue/ to-fatigue: Point in which one can no longer perform.

## Competing interests

The author declares no competing interests and received no financial rewards.

## Author’s contributions

SL conceived the study design; drafted the manuscript; collected the data; analyzed the results; and wrote, read and approved the final manuscript.

## Author’s information

Shannan Lynch completed this study as part of her dissertation requirements at Trident University, 5757 Plaza Drive #100 Cypress, California.

## References

[B1] Kerksick C Harvey T Stout J Campbell B Wilbor C Kreider R Kalman D Ziegenfuss T Lopez H Landis J Ivy JL Antonio J International Society of Sports Nutrition position stand: nutrient timing J Int Soc Sports Nutr 2008 3 5 17 BiMed Central Full Text 18834505 10.1186/1550-2783-5-17 PMC2575187

[B2] Aragon AA Schoenfeld BJ Nutrient timing revisited: is there a post-exercise anabolic window? JISSN 2013 10 5 PubMed Abstract 23360586 10.1186/1550-2783-10-5 PMC3577439

[B3] Jentjens R Jeukendrup A Determinants of post-exercise glycogen synthesis during short-term recovery Sports Med 2003 33 2 117 44 PubMed Abstract 12617691 10.2165/00007256-200333020-00004

[B4] Ebbeling CB Clarkson PM Exercise-induced muscle damage and adaptation Sports Med 1989 7 207 234 PubMed Abstract 2657962 10.2165/00007256-198907040-00001

[B5] Dolezal BA Potteiger JA Jacobsen DJ Benedict SH Muscle damage and resting metabolic rate after acute resistance exercise with an eccentric overload Med Sci Sports Exerc 2000 32 7 1202 1207 PubMed Abstract 10912882 10.1097/00005768-200007000-00003

[B6] Brancaccio P Lippi G Maffulli N Biochemical markers of muscular damage Clin Chem LabMed 2010 48 6 757 767 Full Text 10.1515/CCLM.2010.179 20518645

[B7] Nikolaidis MG Jamurtas AZ Paschalis V Fatouros IG Koutedakis Y Kouretas D The effect of muscle-damaging exercise on blood and skeletal muscle oxidative stress: magnitude and time-course considerations Sports Med 2008 38 7 579 606 PubMed Abstract 18557660 10.2165/00007256-200838070-00005

[B8] Miranda-Vilela AL Akimoto AK Lordelo GS Pereira LC Grisolia CK Klautau-Guimarães MD Creatine kinase MM TaqI and methylenetetrahydrofolate reductase C677T and A1298C gene polymorphisms influence exercise-induced C-reactive protein levels Eur J Appl Physiol 2012 112 3 941 950 ProQuest Full Text 21706313 10.1007/s00421-011-1961-9

[B9] Nakajima T Kurano M Hasegawa T Takano H Iida H Yasuda T Nagai R Pentraxin3 and high-sensitive C-reactive protein are independent inflammatory markers released during high-intensity exercise Eur J Appl Physiol 2010 110 5 905 9013 ProQuest Full Text 20640440 10.1007/s00421-010-1572-x

[B10] Gee TI French DN Howatson G Payton SJ Berger NJ Thompson KG Does a bout of strength training affect 2,000 m rowing ergometer performance and rowing-specific maximal power 24 h later? Eur J Appl Physiol 2011 111 11 2653 2662 ProQuest Full Text 21390543 10.1007/s00421-011-1878-3

[B11] Girard O Mendez-Villanueva A Bishop D Repeated-sprint ability - part I: factors contributing to fatigue Sports Med 2011 41 8 673 94 ProQuest Full Text 21780851 10.2165/11590550-000000000-00000

[B12] Clarkson PM Hubal MJ Are women less susceptible to exercise-induced muscle damage? Curr Opin Clin Nutr Metab Care 2001 4 60 527 531 PubMed Abstract 11706288 10.1097/00075197-200111000-00011

[B13] Sharp CP Pearson DR Amino acid supplements and recovery from high-intensity resistance training J Strength Cond Res 2010 24 4 1125 1130 ProQuest Full Text 20300014 10.1519/JSC.0b013e3181c7c655

[B14] Baty JJ Hwang H Ding Z Bernard JR Wong B Kwon B Ivy JL The effect of a carbohydrate and protein supplement on resistance exercise performance, hormonal response, and muscle damage J Strength Cond Res 2007 21 321 329 ProQuest Full Text 17530986 10.1519/R-21706.1

[B15] Howarth KR Moreau NA Phillips SM Gibala MJ Co-ingestion of protein with carbohydrate during recovery from endurance exercise stimulates skeletal muscle protein synthesis in humans J Appl Physiol 2009 106 1394 1402 Full Text 19036894 10.1152/japplphysiol.90333.2008

[B16] Borg GA Psychophysical bases of perceived exertion Med Sci Sports Exerc 1982 14 5 377 381 PubMed Abstract 7154893

[B17] Borg GA Perceived exertion (Borg rating of perceived exertion scale) [http://www.cdc.gov/physicalactivity/everyone/measuring/exertion.html]

[B18] Beelen M Burke LM Gibala MJ van Loon L Nutritional strategies to promote post-exercise recovery Int J Sport Nutr Exerc Metab 2010 20 6 515 532 Full Text 21116024 10.1123/ijsnem.20.6.515

[B19] Ivy JL Ding A Hwang H Cialdella-Kam LC Post-exercise carbohydrate-protein supplementation: phosphorylation of muscle protein involved in glycogen synthesis and protein translation Amino Acids 2008 35 89 97 ProQuest Full Text 18163180 10.1007/s00726-007-0620-2

[B20] Jentjens RL van Loon LJ Mann CH Wagenmakers AJ Jeukendrup AE Addition of protein and amino acids to carbohydrates does not enhance post-exercise muscle glycogen synthesis J Appl Physiol 2001 91 2 839 846 Publisher Full Text 11457801 10.1152/jappl.2001.91.2.839

[B21] Stephens BR Braun B Impact of nutrient intake timing on the metabolic response to exercise Nutr Rev 2008 66 8 473 476 PubMed Abstract 18667009 10.1111/j.1753-4887.2008.00079.x

[B22] American College of Sports Medicine [ACSM] ACSM’s Guidelines for Exercise Testing and Prescription: Seventh Edition 2005 Philadelphia, PA: Lippincott, Williams, and Wilkins 20

[B23] Baechle TR Earle RW Essentials of Strength Training and Conditioning: Second Edition 2000 Champaign, IL: Human Kinetics 408 409

[B24] Fitday.com [http://www.fitday.com]

[B25] Thomas JR Nelson JK Silverman SJ Research Methods in Physical Activity: Fifth Edition 2005 Champaign, IL: Human Kinetics 152 160

[B26] Smith AE Walter AA Graef JL Kendall KL Moon JR Lockwood CM Fukuda DH Beck TW Cramer JT Stout JR Effects of beta-alanine supplementation and high-intensity interval training on endurance performance and body composition in men; a double-blind trial J Int Soc Sports Nutr 2009 11 6 5 BioMed Central Full Text 19210788 10.1186/1550-2783-6-5 PMC2649036

[B27] Haff GG Koch AJ Potteiger JA Kuphal KE Magee LM Green SB Jakicic JJ Carbohydrate supplementation attenuates muscle glycogen loss during acute bouts of resistance exercise Int J Sport Nutr Exerc Metab 2000 10 326 339 Publisher Full Text 10997956 10.1123/ijsnem.10.3.326

[B28] Boesch C Musculoskeletal spectroscopy J Magn Reson Imaging 2007 25 2 321 338 Full Text 17260389 10.1002/jmri.20806

[B29] Ivy JL Goforth HW Damon BM McCauley TR Parsons EC Price TB Early post-exercise muscle glycogen recovery is enhanced with a carbohydrate-protein supplement J Appl Physiol 2002 93 4 1337 1344 Publisher Full Text 12235033 10.1152/japplphysiol.00394.2002

[B30] Tipton KD Elliott TA Cree MG Aarsland AA Sanford AP Wolfe RR Stimulation of net protein synthesis by whey protein ingestion before and after exercise Am J Physiology Endocrinol Metab 2007 292 71 76 Publisher Full Text 10.1152/ajpendo.00166.2006 16896166

[B31] Hartman JW Tang TE Wilkinson SB Tarnopolsky MA Lawrence RL Fullerton AV Phillips SM Consumption of fat-free fluid milk following resistance exercise promotes greater lean mass accretion than does consumption of soy or carbohydrate in young, novice, male weightlifters Am J Clin Nutr 2007 86 373 381 Publisher Full Text 17684208 10.1093/ajcn/86.2.373

[B32] Wilkinson SB Tarnopolsky MA Macdonald MJ Macdonald JR Armstrong D Phillips SM Consumption of fluid milk promotes greater muscle protein accretion after resistance exercise than does consumption of an isonitrogenous and isoenergetic soy-protein beverage Am J Clin Nutr 2007 85 1031 1040 17413102 10.1093/ajcn/85.4.1031

[B33] Tang JE Moore DR Kujbida GW Tarnopolsky MA Phillips SM Ingestion of whey hydrolysate, casein, or soy protein isolate: effects on mixed muscle protein synthesis at rest and following resistance exercise in young men J Appl Physiol 2009 107 3 987 992 Publisher Full Text 19589961 10.1152/japplphysiol.00076.2009

[B34] Cribb PJ Williams AD Carey MF Hayes A The effect of whey isolate and resistance training on strength, body composition, and plasma glutamine Int J Sport Nutr Exerc Metab 2006 16 494 509 PubMed Abstract 17240782 10.1123/ijsnem.16.5.494

[B35] Cooke MB Rybalka E Stathis CG Cribb PJ Hayes A Whey protein isolate attenuates strength decline after eccentrically-induced muscle damage in healthy individuals JISSN 2010 7 30 Publisher Full Text 20860817 10.1186/1550-2783-7-30 PMC2955583

[B36] Backhouse SH Bishop NC Biddle SJ Williams C Effect of carbohydrate and prolonged exercise on affect and perceived exertion Med Sci Sports Exer 2005 37 10 1768 1768 Full Text 10.1249/01.mss.0000181837.77380.80 16260979

[B37] Backhouse SH Ali A Biddle SJ Williams C Carbohydrate ingestion during prolonged high-intensity intermittent exercise: impact on affect and perceived exertion Scand J Med Sci Sports 2007 17 5 605 610 PubMed Abstract 17316376 10.1111/j.1600-0838.2006.00613.x

[B38] Kalman DS The effects of feeding protein as compared to carbohydrate or the two combined on athletic performance, perceived exertion and biochemical markers of anabolism and catabolism in trained athletes under glycogen depleted conditions PhD dissertation 2007 Trident University, Department of Health Sciences ProQuest Full Text

[B39] Utter AC Kang J Robertson RJ Nieman DC Chaloupka EC Suminski RR Piccinni CR Effect of carbohydrate ingestion on ratings of perceived exertion during a marathon Med Sci Sports Exer 2002 34 11 1779 1784 PubMed Abstract 10.1097/00005768-200211000-00014 12439083

[B40] Utter AC Kang J Nieman DC Vinci DM McAnulty SR Dumke CL McAnulty L Ratings of perceived exertion throughout an ultramarathon during carbohydrate ingestion Percept Mot Skills 2003 97 1 175 184 PubMed Abstract 14604037 10.2466/pms.2003.97.1.175

[B41] Utter AC Kang J Nieman DC Dumke CL McAnulty SR Vinci DM McAnulty LS Carbohydrate supplementation and perceived exertion during prolonged running Med Sci Sports Exerc 2004 36 6 1036 1041 Full Text 15179174 10.1249/01.mss.0000128164.19223.d9

[B42] Utter AC Kang J Nieman DC Brown VA Dumke CL McNulty SR McNulty LS Carbohydrate supplementation and perceived exertion during resistance exercise J Strength Cond Res 2005 19 4 939 944 ProQuest Full Text 16287372 10.1519/R-16994.1

